# Technical and biological variance structure in mRNA-Seq data: life in the real world

**DOI:** 10.1186/1471-2164-13-304

**Published:** 2012-07-07

**Authors:** Ann L Oberg, Brian M Bot, Diane E Grill, Gregory A Poland, Terry M Therneau

**Affiliations:** 1 Division of Biomedical Statistics and Informatics, Department of Health Sciences Research, Mayo Clinic, 200 1st St SW, Rochester, MN, 55905, USA; 2 Mayo Vaccine Research Group, Mayo Clinic, 200 1st St SW, Rochester, MN, 55905, USA; 3 Statistical Genetics, Sage Bionetworks, 1100 Fairview Ave N, M1-C108, Seattle, WA, 98109, USA; 4 Program in Translational Immunovirology and Biodefense, Mayo Clinic, 200 1st St SW, Rochester, MN, 55905, USA; 5 Department of Medicine, Mayo Clinic, 200 1st St SW, Rochester, MN, 55905, USA

## Abstract

**Background:**

mRNA expression data from next generation sequencing platforms is obtained in the form of counts per gene or exon. Counts have classically been assumed to follow a Poisson distribution in which the variance is equal to the mean. The Negative Binomial distribution which allows for over-dispersion, i.e., for the variance to be greater than the mean, is commonly used to model count data as well.

**Results:**

In mRNA-Seq data from 25 subjects, we found technical variation to generally follow a Poisson distribution as has been reported previously and biological variability was over-dispersed relative to the Poisson model. The mean-variance relationship across all genes was quadratic, in keeping with a Negative Binomial (NB) distribution. Over-dispersed Poisson and NB distributional assumptions demonstrated marked improvements in goodness-of-fit (GOF) over the standard Poisson model assumptions, but with evidence of over-fitting in some genes. Modeling of experimental effects improved GOF for high variance genes but increased the over-fitting problem.

**Conclusions:**

These conclusions will guide development of analytical strategies for accurate modeling of variance structure in these data and sample size determination which in turn will aid in the identification of true biological signals that inform our understanding of biological systems.

## Background

Next generation sequencing is a tool that is revolutionizing scientific research with its unprecedented depth of coverage, accuracy, precision, and the ability to link gene expressions with phenotype. The Illumina Genome Analyzer (GA), originally by Solexa, enables interrogation of mRNA expression via the mRNA Sequencing protocol. There are several reports on quality assessments of next generation sequencing and comparisons with microarray gene expression [1,2]. Biological signal has been evaluated in single biological replicates of cell lines or tissue samples from this platform [3-5]. Anders and Huber report on variation in pools of two fruit fly embryos [6]; however, to date there is no thorough report on the functional form of variance in variability in mRNA Sequencing data from true human biological replicates. Thus, we evaluated the structure of biological variability and statistical modeling strategies useful for determining differential expression in mRNA-Seq data with true biological replicates.

First we describe some distributional background. The Poisson distribution is commonly assumed when modeling count data. This distribution considers each individual piece of mRNA to be a random draw from a large collection of pieces of mRNA with some probability vector describing the relative proportion across all possible mRNA pieces. A piece could refer to a particular exon or gene according to the researcher’s interest. The Poisson distribution appears to describe well the variation observed between two technical replicates of the same specimen, i.e., two aliquots of the same library allocated to two lanes on a flow cell [3-5]. The mean, μ, and variance are expected to be equal when sampling from a Poisson distribution, i.e., Var(y) = μ.

Biological replication adds another level of variability to the observed data. Biological variability is that due to inter-individual differences between human or animal subjects, for example, which cause the probability vector describing the distribution of mRNA strands to differ between subjects. Thus, when count data are observed in multiple biological replicates, the observed variance is a sum of both the technical and biological variability pieces. This results in the observed variance being larger than expected under the Poisson distribution. That is, the variance is larger than the mean. This scenario is termed “over-dispersion” [7].

In the simplest case, variance increases as a linear function of the mean, i.e., the variance is a constant multiplied by the mean, Var(y) = kμ. We denote this as the over-dispersed (OD) Poisson throughout and model parameters can be estimated via quasi-likelihood methods. A more sophisticated model assumes the within-specimen (technical) variation follows a Poisson distribution and the between-specimen mean values follow a gamma distribution. This gives rise to the negative binomial (NB) distribution in which the variance increases as a quadratic function of the mean, i.e., Var(y) = μ + φμ^2^[7].

Our goal in the present work was to characterize the mean-variance relationship in mRNA Seq data in order to guide the choice of distributional assumptions. We first evaluated technical variability in gene-level counts to ensure consistency with what others have reported. Next, we evaluated the variance structure between biological replicates within a treatment group, considering the functions Var(y) = μ, kμ, and μ + φμ^2^ with and without normalization and blocking factors. We believe this work will be useful to others in analyzing and interpreting similar data.

## Methods

### Subjects

Twenty five study subjects representing the extremes of the humoral immune responses to rubella vaccine (12 high antibody responders with a median titer of 145 IU/mL and 13 low responders with a median titer of 10 IU/mL) were selected from a large population-based, age-stratified random sample of 738 healthy children and young adults (age 11 to 19 years), from Olmsted County, Minnesota. Clinical and demographic characteristics of the population based sample have been previously reported [8]. This population-based candidate gene association study was performed to assess the importance of single nucleotide polymorphisms (SNPs) and genes involved in the immune response heterogeneity to rubella vaccine [8-10]. All study participants had been previously immunized with two doses of measles-mumps-rubella (MMR-II) vaccine with a median time since last immunization to enrollment (blood draw and measurement of antibody levels) of 5.8 years. The Mayo Clinic Institutional Review Board granted approval for the study. Written, informed consent and assent from subjects and/or parents/guardians was obtained at the time of enrollment.

### PBMC culture, stimulation and RNA isolation

Subjects’ PBMC (peripheral blood mononuclear cells) were thawed and stimulated (or left unstimulated) with live rubella virus (multiplicity of infection, MOI = 5, 48 hrs). Total RNA was extracted from stabilized cells using RNeasy Plus mini kit (Qiagen, Valencia, CA). RNA concentration and quality were assessed by Nano Chip kit analysis on an Agilent 2100 Bioanalyzer (Agilent, Palo Alto, CA). Fifty samples from 25 subjects were completed for culture, RNA extraction and RNA quality control with adequate concentration and purity (lack of DNA contamination), as well as good RNA integrity and lack of RNA degradation.

### Library preparation and sequencing

Libraries were prepared using the mRNA-Seq Sample Prep Kit (Illumina, San Diego, CA) following the manufacturer’s instructions. Briefly, polyadenylated RNA was isolated from 1 μg of total RNA using two rounds of hybridization to oligo-dT magnetic beads. The mRNA samples were chemically fragmented, reverse transcribed and converted into double stranded cDNA. Unique Illumina adaptors were ligated to the DNA fragments after end repair (to produce blunt ends) and A-base tailing. Fragments of approximately 200 bp were gel purified and amplified by PCR. The libraries were validated and quantified on an Agilent 2100 Bioanalyzer (Agilent, Palo Alto, CA) using DNA 1000 Chip kits. Sequencing was carried out on the Genome Analyzer GAIIx (Illumina, San Diego, CA). Samples were sequenced as single end reads using Illumina’s Single Read Cluster Generation kit (v2) and 51 Cycle SBS Sequencing Kit (v3) following the manufacturer’s instructions.

The first five flow cells were processed using Sequencing Control Studio (SCS) v 2.01 and the last eight flow cells were processed with SCS v 2.4 which allowed for higher cluster densities and higher pass filter rates. The images from the sequencing cycles were processed using the Illumina Pipeline Software v1.5. Specifically, images were converted to signal intensities using Illumina Pipeline’s FireCrest program. Base calling from intensity values was performed, and the quality scores for every base were calculated using the Bustard program. Illumina’s alignment tool, ELAND, was used to align the sequence reads to genome build 36 and exon junction databases. Illumina’s CASAVA tool version 1.0 was used to summarize the alignment results using only reads that mapped to a unique genome location and CASAVA results were imported into Genome Studio to generate the count tables for genes, exons, and exon-junctions. Sequencing pass/fail quality was determined by cluster densities, percent of clusters passing filters, percent of reads aligning to the reference, and percent error rate of the alignment.

### Statistical experimental design

**Figure 1:**
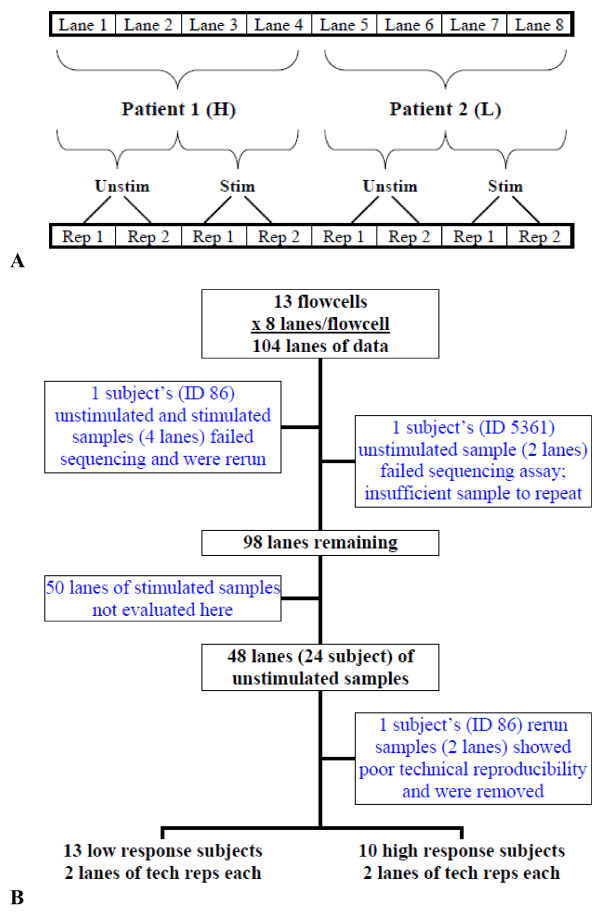
**Study design. A**) Cartoon depicting the allocation of subject samples to flow cells. One high (H) and one low (L) responder was allocated to each flow cell. Within a flow cell, each patient was randomly allocated to lanes 1-4 or 5-8, ensuring that H/L response was balanced over lanes across all flow cells. Finally, for each subject, two technical replicates of their stimulated and unstimulated specimens were randomly allocated to the first two or second two lanes such that stimulation status was balanced over lanes. **B**) Flow diagram demonstrating the full initial sample set and reasons for excluded lanes of data for the final analysis data set.

Specimens were randomly allocated to flow cells and lanes as follows (Figure 1A). First, one high and one low responder were allocated to each flow cell (two subjects total per flow cell), ensuring that flow cell and response group were not confounded. Within a flow cell, the high and low responders were randomly assigned to lanes 1-4 or 5-8 such that response status was balanced over lanes, ensuring that lane and response group were not confounded. Finally, for each subject, two technical replicates each of their stimulated and unstimulated specimens were randomly allocated to the first two or second two lanes such that stimulation status was balanced over lanes, ensuring that lane and stimulation status were not confounded.

### Statistical methods

The endpoint used for analysis was total reads or counts per gene; for the analysis of technical variation this is counts per lane while for biological variation this is counts summed over two technical replicate lanes. We evaluated the suitability of the Poisson, OD Poisson, and NB assumptions for modeling biological variability. The Poisson distribution assumes variance is equal to the mean *μ*. The OD Poisson assumes the variance increases as a linear function of the mean, *kμ*, where *k* is a constant and *μ* is the true mean. The NB distribution assumes the variance increases as a quadratic function of the mean as *μ + φμ*^*2*^, where *φ* is a constant.

Model Goodness-of-fit (GOF) was assessed via quantile-quantile (QQ) plots of per-gene Pearson statistics ${Χ}^{2}=\sum_{i=1}^{n}{\left(y_{i}-{\hat{y}}_{i}\right)}^{2}/Var\left({\hat{y}}_{i}\right)$ as has been done previously [4]. Here, *i* indexes *n* samples, *y* is the observed count, ${\hat{y}}_{i}$ is the predicted value and $Var\left({\hat{y}}_{i}\right)$ is the appropriate variance of the predicted value (${\hat{y}}_{i}$ for the Poisson, $\hat{k}{\hat{y}}_{i}$ for the OD Poisson and ${\hat{y}}_{i}+\hat{\phi }{\hat{y}}_{i}^{2}$ for the NB). This statistic is asymptotically chi square with *n-p* degrees of freedom, where *n* is the sample size and *p* is the number of model parameters. While GOF statistics are a rather crude measure of how well a model fits, the magnitude of changes observed here are informative. We verified that the asymptotic assumptions were reasonable with the present sample size in simulated NB data (Additional file 1: Figure S1). While the empirical distributions do not have values quite as extreme in the right tail as theoretical distributions, the deviation from the identity line is very small relative to the distributional changes observed herein. An alternative to the Pearson statistic is residual deviance, but this statistic was found to be ill behaved, even in simulated data. Such behavior has been documented previously [7].

The generalized linear model (GLM) framework was used to fit per-gene models to test for differential expression between high and low response groups using the log link function [7]. The most basic model included a term for high/low response group. These were fit to unstimulated specimens only so as to focus on biological variation in the absence of correlation between paired specimens. Counts for the two technical replicate lanes (which we call lane-pair) were summed for these models. Thus, these fits had *p* = 1 model degree of freedom.

Model fits were evaluated with no normalization, with total count per lane-pair, or with 75^th^ percentile count per lane-pair as a normalization constant [5]. Normalization constants were included as an offset term in the GLM model, i.e., a term with the coefficient forced to be 1.0. The normalization offset leads to the interpretation of the gene counts as a rate, e.g., the portion of counts out of the total lane counts [11]. In addition, fits were evaluated with no blocking factor or with categorical variables added to the model to indicate flow cell (*p* = 13), lane-pair (*p* = 4) or library preparation batch (*p* = 6) as blocking factors. These blocking factors are potential sources of systematic non-biological variation in the experiment. There may be global shifts in counts due to flow cell, lane position on the flow cell such as middle versus edge, or PCR batch effects. Lastly, per-gene, local and global variance parameter estimation strategies were evaluated.

All statistical computing was performed in R [12]. The glm function in R was used to fit all models owing to the flexibility in modeling biological and experimental effects. Models based upon NB distributional assumptions were fit with either per-gene, moderated or global estimates of *φ*. Per-gene estimates were estimated within the glm.nb function call in the MASS package on a per-gene basis. Global and local (moderated) estimates of φ were computed with the edgeR package [13-16] in R. In that package, the global estimate is computed via quantile-adjusted conditional maximum likelihood [15] and is shared by all genes. The local estimate is an approximate empirical Bayes estimate which shrinks per-gene estimates towards the common global estimate [14]. The degree of shrinkage for local estimates depends on the prior.n parameter, where larger values result in more shrinkage towards the global value. We evaluated setting prior.n equal to 3, 10 (the default value), and 20; since a prior.n of 3 yielded much better fits than 10 or 20 (data not shown), results are presented here with prior.n = 3.

Convergence rates were excellent for all models, with the worst case being non-convergence for four genes when a blocking factor was included in the model. The data have been deposited in the Gene Expression Omnibus and are available (with anonymous gene names since the biological findings have not yet been published) via the following link: http://www.ncbi.nlm.nih.gov/geo/query/acc.cgi?token=nxahvuioqmkwwzg&acc=GSE29022.

## Results

### Subjects and assays

All 25 study subjects were female Caucasians to minimize variation. Median rubella-specific antibody response in the 12 high response subjects was 145 IU/ml (min 115, max 325) and in the 13 low response subjects was 10 IU/ml (min 3, max 14) [8].

Figure 1B contains a schematic of the attempted and failed assays. Sample libraries were prepared in seven PCR batches and a total of thirteen flow cells were used. Sequencing failed for all four lanes of subject 86 and for the unstimulated lanes of subject 5361 and we did not receive data for these lanes. For subject 86, the libraries were determined to be of good quality and were re-sequenced, filling the eighth flow cell; for subject 5361 the libraries were not of good quality and there was not sufficient sample to repeat the library preparation. Since the focus of this manuscript is on the mean-variance relationship in these data in the absence of correlation, we focus here on the 48 lanes of data corresponding to the unstimulated specimens from 24 subjects for simplicity.

**Figure 2:**
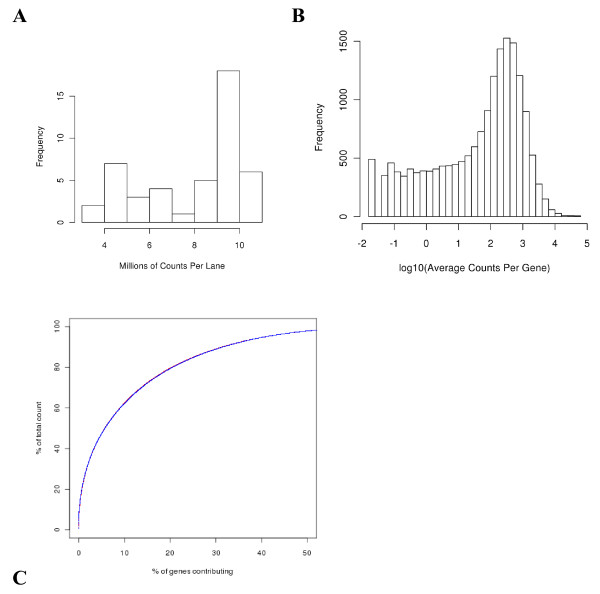
**Distributions of counts. A**) Histogram of total reads per lane for 46 lanes (unstimulated specimens) on the scale of millions of reads. **B**) Frequency histogram of average counts per gene per lane on the log10 scale. **C**) Cumulative percent of average counts per lane as a function of the percent of genes contributing. Lines for both high (red) and low (blue) responders were drawn, but not distinguishable.

At least one count was observed for 17,337 genes. Total counts per lane ranged from 3.7 million to 10.7 million (Figure 2A). The wide range was due to a software upgrade midway through the study. The distribution of counts/gene/lane spanned five orders of magnitude as demonstrated in Figure 2B. Sixty-two percent of the total counts were from 10% of the genes, in both the high and low responders (Figure 2C).

### Technical variation

We assessed technical variation as the variation between the same specimen pipetted onto two lanes of a flow cell. Technical reproducibility in these data was good as demonstrated by scatter and minus versus average (MVA) plots (Additional file 1: Figure S2). Once scaled for total lane count, virtually no linear bias was detected as demonstrated by the grey smoother nearly overlaying the y = 0 line. In addition, there was no hint of any nonlinear biases as is often seen in gene expression microarray data. This was true for all pairs of technical replicates.

QQ plots assuming Poisson-type variation were made for all pairs of technical replicates using Pearson GOF statistics; all 24 are available in Additional file 1: Figure S3. For Poisson-type variation, the points would be expected to fall on the identity line. Eighteen of the plots demonstrated variation very close to expected. Three of the plots demonstrated a moderate fit of the Poisson distribution with observed Pearson quantile scales ranging from approximately 25 to 50 indicating larger than expected variance. Two plots demonstrated poor fit overall as evidenced by large observed Pearson quantile scales, one reaching approximately 250 and the other approximately 1,200. The observed Pearson quantiles for the technical replicates from subject 86 reached approximately 25,000. As there were problems with this sample in the laboratory, and because the distribution of GOF statistics was so far from expected and so different from the others, we discarded this sample from further analyses. Similar to what others have found, we concluded that in general, technical variation followed a Poisson distribution with variance equal to the mean. Similar results for technical variation were found in the stimulated specimens (data not shown). With this conclusion we summed the per-gene counts from the two technical replicates for further analyses as in [4].

### Biological variation

We define biological variation as the variation between multiple subjects in the same group, e.g., high versus low responders, cancer versus normal, treatment versus not, etc. This variation may be small for cell lines and moderate for genetically identical animals, but can be very large for human samples. Analysis methods need to be able to cope with and appropriately model this variation.

**Figure 3:**
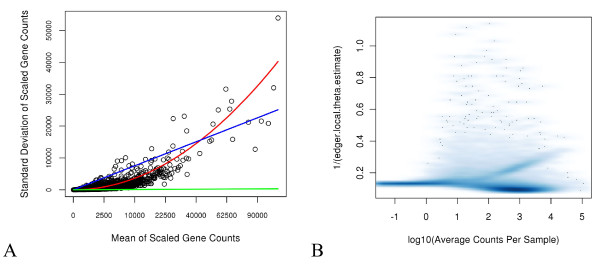
**Assessing presence and magnitude of over-dispersion. A**) The horizontal axis indicates the mean scaled count within each of the high/low response groups on the square root scale (labeled on the raw scale) and the vertical axis indicates the variation on the standard deviation (i.e. square root of the variance) within each group. Each gene is thus represented by two points, one for each response group. The green line corresponds to the Poisson assumptions, the blue line corresponds to OD Poisson assumptions, and the red line corresponds to NB assumptions, with lines constructed as described in the text. **B**) Local estimates of *φ* from the edgeR function versus per-group mean count. The shading indicates density of points in that area with darker shading representing higher density.

**Figure 4:**
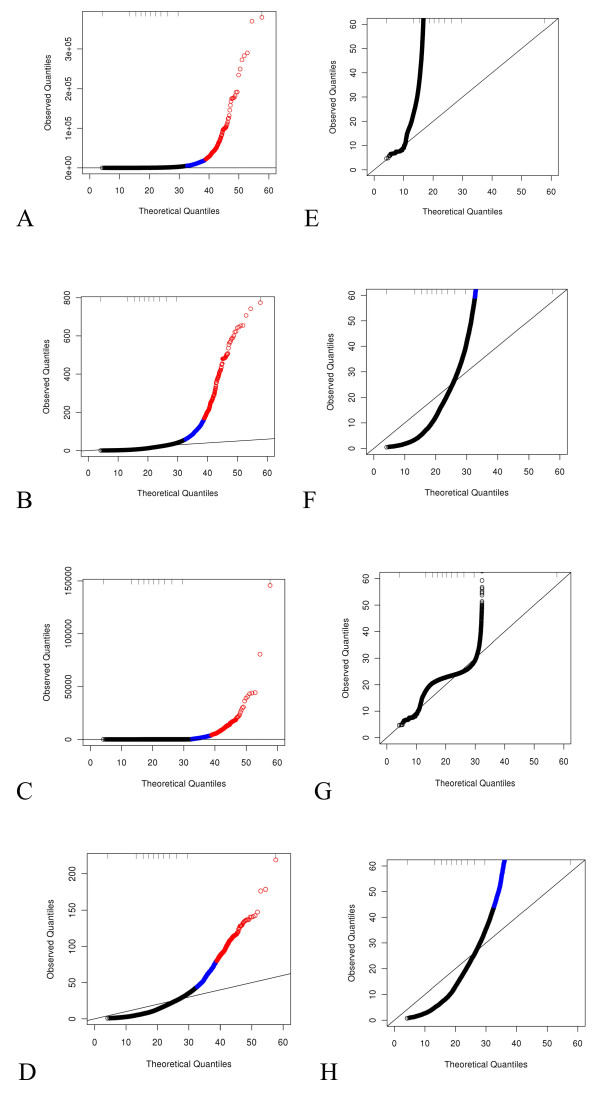
**Distribution of GOF statistics.** Residual QQ plots of model fits normalized with the 75% count and no blocking factor. Tick-marks along the top indicate deciles. The top 5% of GOF statistics are indicated in alternate colors with the top 1% being red and the next 4% being blue. **A**) Standard Poisson, **B**) NB with a global estimate of *φ*, **C**) NB with per-gene estimates of *φ*, **D**) NB with local estimates of *φ*. Panels **E**-**H** are as in A-D but zoomed in on the bottom left corner of the plots.

In order to understand the functional form of the mean-variance relationship, we created a scatter plot of the sample biological variation $S^{2}$ versus the sample mean $\bar{x}$, both calculated within high and low response groups separately so as to estimate subject-to-subject variability. Note that in this experiment, this variance will include variation due to flow cell as well since there is only one subject per response group per flow cell. Three lines were overlaid on the points: i) an identity line representing Poisson assumptions, ii) a linearly increasing line with slope estimated from a regression of $S^{2}=k\bar{x}$ (no intercept) representing OD Poisson assumptions, and iii) a quadratic function estimated from a regression of $S^{2}=\bar{x}+\phi {\bar{x}}^{2}$ representing NB assumptions (the slope estimate was $\hat{\phi }=0.178$). Here we show this plot on the standard deviation scale for ease of viewing, and with the global estimate of $\hat{\phi }=0.131$ as estimated via the edgeR function [16] in R over all genes (Figure 3A, R function to produce 3A provided in Additional file 2, plotMeanVariance.R). Unlike an identity or linear over-dispersion function, the quadratic variance function appeared to describe the mean-variance relationship well for the majority of the genes. As a comparison of the effect on goodness of fit, QQ plots from models fit based on standard Poisson and NB assumptions are provided (Figure 4A, E; R function to produce QQ plots such as those in Figure 4 is provided in Additional file 3, fitNBedgeR.R). Consistent with the conclusions based on Figure 3A, the QQ plot based on NB assumptions with φ fixed at the edgeR global estimate of 0.131 demonstrated dramatically improved fit overall with the observed Pearson quantiles extending to approximately 800 (Figure 4B, F), much less than the 300,000 for Poisson assumptions.

We questioned whether a one-size-fits-all variance structure was appropriate. It is biologically plausible (and many would argue likely) that the relationship, i.e. the precise multiplier *φ*, varies between genes. Thus, models were fit estimating over-dispersion on a per-gene basis assuming a quadratic functional form. Note that the quantities plotted in QQ plots based on the linear over-dispersion assumption are used in the estimation of the over-dispersion parameter in the R function and therefore by definition were not useful for assessing model fit. The QQ plot based on NB distributional assumptions with per-gene estimates of *φ* demonstrated worse fits than when the global estimate was used with observed Pearson quantiles extending up 150,000 for all genes, with GOF statistics for all but two genes below 50,000 (Figure 4C, G). To investigate further, we exported local estimates of *φ* from the edgeR function and held these fixed. These fits demonstrated improvement over those based on the global estimate of *φ* with a maximum observed Pearson Chi-squared value of approximately 220 (Figure 4D, H). Eighty-two percent of the local *φ* estimates were less than 0.15 (Figure 3B). Note that a *φ* of 0 would correspond to no over-dispersion, consistent with Poisson distributional assumptions. Approximately 5% of the genes had an estimate of *φ* greater than 0.25. Upon investigation of the genes with *φ* estimates in the spike of density beginning at a log_10_(average count per lane-pair) of approximately 2 and extending to approximately 4.5 on the horizontal axis (Figure 3B), we found that both replicates from one patient sample had 0 counts for all of these genes. The data from this subject otherwise appear similar to that from other subjects. This phenomenon will be investigated further. Ignoring this spike, the estimates of *φ* do not appear to be a function of the mean in these data. We focus on the quadratic mean-variance functional form with local estimates from here forward for four reasons. First, a quadratic function fits the global mean-variance relationship for biological variation (Figure 3A). Second, the biological interpretation of the NB distribution is appropriate; count data that follow a Poisson distribution conditional on a subject-specific mean, together with between-subject means that follow a Gamma distribution, give rise to the NB distribution. Third, it was clearly difficult to estimate the mean-variance relationship on a per-gene basis (Figure 4). Fourth, we do not believe that all genes must share the same mean-variance relationship.

### Experimental variation

As in Bullard et al. [5], we found that choice of normalization constant had little effect on GOF distributions (data not shown). Therefore we present results taking the 75% count as the normalization factor since Bullard et al. found this normalization scale to have less bias in fold-change estimates and better sensitivity and specificity than none or total lane count. The rationale for using the 75% count to adjust for varying sequencing depths rather than, say, the total lane count, is to avoid the possibility of a high-count gene that is differentially expressed being overly influential on the normalization [5,17].

**Figure 5:**
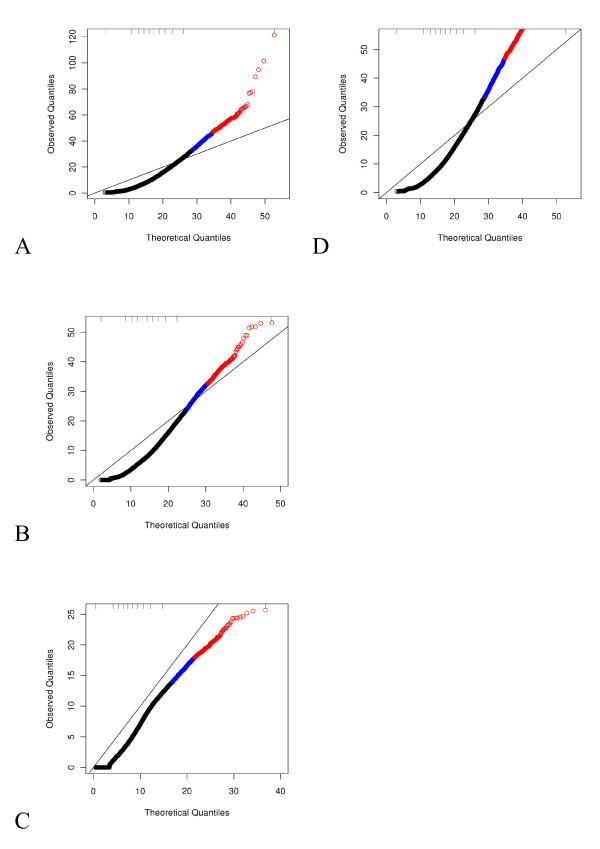
**Distribution of GOF statistics when experimental factors are included in the model.** QQ plots of model fits with the NB distribution, local estimates of *φ* and 75^th^ percentile count offset including blocking factors as indicated. Tick-marks along the top indicate deciles. The top 5% of GOF statistics are indicated in alternate colors with the top 1% being red and the next 4% being blue. **A**) lane-pair, **B**) library preparation batch, **C**) flow cell. Panel **D** is the same as A, but zoomed in on the bottom left corner of the plot; no zoom is needed for panels B and C.

**Figure 6:**
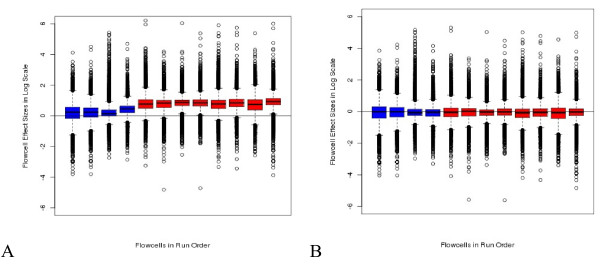
**Distribution of flow cell effects.** Box plots of contrast coefficient estimates indicating the difference of flow cells 2 – 13 from flow cell 1 sorted by run order. The flow cells represented by the left four (blue) boxes were analyzed with SCS v 2.01 while the right-most eight (red) were analyzed with SCS v 2.4. **A**) Results from models without an offset to account for differences in total counts per lane. **B**) Results from models including the 75^th^ percentile offset.

Potential sources of experimental variation examined here were flow cell, lane-pair and library preparation batch and all of these resulted in lower maximum observed Pearson chi-square values which can be seen on the QQ plots (Figure 5). Adding one of lane-pair, library preparation batch, or flow cell to the model reduced the maximum observed Pearson chi-squared value to about 120, 50 or approximately 25, respectively. The distribution for the library preparation batch was the closest to the expected distribution of the three. Addition of flow cell to the model caused the entire distribution of GOF statistics to be smaller than expected. The largest obvious difference between flow cells was due to the software upgrade mid-way through the experiment. Indeed, when models were fit without a normalization offset term, the shift in the distribution of flow cell effect estimates corresponded with the date of the SCS software upgrade mid-way through the study which increased the number of reads by several million per lane (Figure 6A). However, when the total or 75^th^ percentile offset was included, there was no clear relationship between flow cell effect estimates and run order (Figure 6B). From this and the distributions of GOF statistics we concluded that something more than just a difference in lane counts could be attributed to flow cell differences. These conclusions were similar regardless of normalization strategy or over-dispersion estimation strategy (data not shown).

While it is important for a model to explain all sources of variation, a balance must be made between this and over-fitting the data. This will especially be the case for studies with extremely small samples which are typically employed for next generation sequencing studies given the intense monetary and time resource utilization.

### Characterizing genes with poor model fit

**Figure 7:**
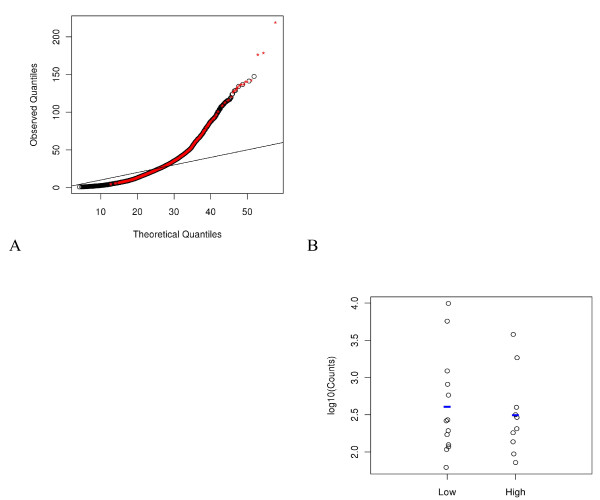
**Understanding causes of poor model fit. A**) GOF statistics for genes with an average count per subject <5 are shown in red on the QQ plot from NB, locally estimated variance with no blocking factor models. **B**) Dot plot demonstrating the large variance in a gene with an extremely large GOF statistic.

We investigated whether the genes with small counts were those with the smallest (indicative of over-fitting) or largest (indicative of under-fitting, or not explaining enough variation) GOF statistics when using NB distributional assumptions with local variance estimate and no blocking factors**.** Interestingly, filtering out low count genes, even up to a total count of 10,000 (average count 435), had only a minor impact on the distributions (data not shown). The GOF statistics for genes averaging less than 5 counts per subject were distributed throughout the range of GOF statistics (red points on Figure 7A). Further inspection of the data records revealed two main scenarios in those genes with the smallest GOF statistics. In the first scenario, the genes had all zero counts in one response group versus non-zero counts in the other response group. For genes in the second scenario, counts within both response groups were very consistent and had very small variance. Data records for those genes having the largest GOF statistics had very large variance. The distributions of counts within the high/low response groups for one such gene had counts spanning over two orders of magnitude in both study groups, i.e., very large variance (Figure 7B).

## Discussion

We set out to evaluate technical, biological, and experimental variation in gene expression measured by mRNA-Seq counts from n = 25 subjects. Technical variation in these data were in keeping with Poisson distributional assumptions in general as has been reported by some [3-5], while others have reported greater than Poisson variation for low counts [18]. The biological variance across all observed genes was over-dispersed relative to the mean, increasing as a quadratic function of the mean. This has important implications for study planning in terms of power and sample size estimation as well as choice of statistical modeling procedures to detect differential expression. Differential expression results were not included here since the focus is on the mean-variance relationship.

Over-dispersion, i.e., variance larger than the mean, is a common phenomenon in observed count data [7]. Others have suggested that over-dispersion in mRNA-Seq data will likely be observed in mRNA-Seq biological replicates and have suggested models relying on OD Poisson or NB assumptions to account for this [5,6,13,16,19]. There was clear evidence of over-dispersion from the standard Poisson distribution in our data. A quadratic function described the mean-variance relationship across all genes better than a linear function, which is consistent with the NB family of distributions. The interpretation of the NB distribution as counts from several within-subject Poisson distributions, each subject having their own mean parameter makes biological sense as well in the study at hand. In μ + φμ^2^, the square root of φ corresponds to the subject-to-subject CV of the Gamma distributed Poisson mean parameters. The CV corresponding to the global estimate of φ for the data presented herein is 36%. We have observed a CV of approximately 9% for technical variation in genetically similar rats and 22% for biological replicates in genetically similar rats (data not shown). This is in keeping with the expectation that biological variation is larger than technical variation, and human variation is larger than genetically inbred animal model variation.

There are several potential sources of experimental variation in these studies including PCR library preparation batch, flow cell, lane, and for large studies, machine and software upgrades. Some effects can be addressed through normalization while others must be modeled directly. We found that normalization strategy had little impact on GOF statistics. Bullard et al. [5] reported similar results and found that the 75% count normalization resulted in nearly no bias and the best sensitivity and specificity without increasing variance. Our data was similar to theirs in that a small percent of the genes contributed a large portion of the counts. Thus, we chose to use the 75% count as the normalization scale in our models. Others have evaluated normalization strategies beyond this [17,20].

Bullard et al. found experimental variation due to library PCR preparation batch to be greater than that due to flow cell [5]. We found that inclusion of library PCR batch, lane-pair and flow cell each resulted in a dramatic reduction in the large GOF statistics resulting in the GOF statistics being too small (i.e., less than expected) in the majority of genes. The GOF statistics were reduced to the extent that, in this particular experiment with only one subject per treatment per flow cell, we believe these terms should not be included in the differential expression models in order to avoid over-fitting. Note that this experiment is an incomplete block design with respect to subject, in which case there is not complete recovery of all effects. The discussion of incomplete recovery of effects has spanned decades in the statistical literature. The classic text by Scheffe points to the use of random effects as a compromise between the extremes of not including the effects or including them as fixed effects [21]. Since estimation in a NB model of both the dispersion parameter and random effects is not straightforward, the experimental effects were modeled as fixed effects for the purposes of this investigation.

Estimation of the over-dispersion parameter has been the subject of much research; see for example the summary in [15]. While the edgeR local estimates of over-dispersion resulted in the most reasonable GOF distributions, the results herein point to the need for further investigation in the estimation of variance for these data. Biologically it makes sense for the variance to differ across genes, and that the mean-variance relationship may vary across genes as well. For example, some systems in the body such as pH are very tightly controlled with dramatic consequences for deviations from the baseline normal levels. Other systems in the body such as cholesterol are less tightly controlled, and allowed to vary widely before consequences are observed. However, with small sample sizes it is likely too difficult to estimate the mean-variance relationship accurately and precisely on a per-gene basis. Local or per-gene estimates of variance are common practice in gene expression microarray and shotgun proteomic literature [22-24]. Anders and Huber recently presented data from two pools of fruit fly embryos in each of two study groups that agree with this assumption and they described a model for variation based on NB distributional assumptions with a “shot noise” portion and a “raw variance” portion [6]. Alternatively, the fact that these model fits result in smaller than expected GOF statistics for a majority of genes may point to the need for a linear variance function for some genes and quadratic variance function for others due to biological phenomenon such as alternative splicing. Indeed, such a modeling strategy has been proposed [25]. It is difficult to compare per-gene appropriateness of OD Poisson and NB distributional assumptions due to the poor behavior of the residual deviance statistic and the fact that estimation of the OD Poisson dispersion parameter is a function of the Pearson statistic. Application of a power transformation to the data in order to use Poisson modeling assumptions has recently been proposed [17]. We expect continual improvement in informatics mapping algorithms to result in decreased variance; e.g. including reads that map to multiple locations on the genome has been shown to improve technical reproducibility [26]. Robust GLMs based on M-estimators may be an attractive alternative as well. A Gaussian approximation to the Poisson distribution is said to be valid when the mean is ≥10 [27]; thus linear models which account for the mean-variance relationship, e.g. via weighted least squares, may be an attractive alternative since model fitting and assessment of model fit is more straightforward than for GLMs.

Our study had several strengths and limitations. We reported on technical and biological variation in mRNA-Seq data from a relatively large sample set and provide valuable insight into biological variation that will be useful to many researchers. The data herein were from true human biological replicates rather than contrived cell line replicates. We expect that the conclusion of a quadratic mean-variance relationship for biological replication will extend to other experimental settings. However, the precise estimates of over-dispersion are expected to be study-specific; it is plausible they would be smaller in inbred mouse models, smaller yet in cell lines. The present study was not designed to assess bias in estimating gene expression, fold-changes or sensitivity and specificity of fold-change detection. While the modeling strategy utilized does not account for the degrees of freedom used in estimating the dispersion parameter, thousands of data points are used in this estimation, so the estimate should be very stable and precise [14]. Unfortunately there was a software upgrade mid-way through the study. Technology in the field of next generation sequencing is progressing at a rapid pace; indeed, data from third generation sequencing is already available. This rapid pace makes upgrades, whether in software or hardware, difficult to avoid in large studies making it important to utilize randomization and blocking when determining assay processing order.

## Conclusions

We found that the within-gene variance structure is over-dispersed relative to the Poisson distribution. As a result, hypothesis tests based on Poisson distributional assumptions will be too liberal (reject more often than they should) and power estimates based on these assumptions will be over-estimated for most genes, i.e., sample sizes will be too small for the estimated power. Local estimates [14-16] of the mean-variance relationship are likely best, but further research is needed to understand the optimal variance estimation strategy. The variance increasing as a function of the mean has implications on modeling strategy even if the data are modeled as continuous values rather than counts; e.g. weighted least squares. Modeling experimental factors within the GLM framework likely requires larger sample sizes. These conclusions will guide development of analytical strategies useful in study planning, accurate modeling of these data, and will aid in the identification of true biological signals that inform our understanding of biological systems. In addition, as with other high dimensional expression assays [19,28-31], since we do not yet thoroughly understand experimental variation in next generation sequencing platforms, it is vitally important to incorporate the fundamental principles of statistical experimental design, namely biological replication, blocking and randomization, during the study design phase in order to avoid confounding of experimental and biological signals.

## Competing interests

The authors wish to declare that GAP is the chair of a data safety monitoring board for Merck for non-rubella novel vaccines in clinical trials.

## Authors’ contributions

ALO helped design the sequencing study, conceived the statistical questions, directed the statistical work and wrote the manuscript. BMB helped refine the study questions, performed all computing and participated in critical interpretation of the data. DEG helped design the sequencing study, helped direct the statistical computing, helped refine the study questions and participated in critical interpretation of the data. GAP helped design the sequencing study. TMT participated in critical interpretation of the data. All authors read and approved the final manuscript.

## Supplementary Material

Additional file 1 **Figure S1.** Evaluation of asymptotic GOF distributional assumptions. QQ plot of GOF statistics from simulated null (i.e., no differential expression) NB data. Data for genes were simulated with mean equal to the mean vector in the unstimulated data presented herein, dispersion parameter equal to the edgeR estimated moderated dispersion parameter values. GOF statistics were calculated for each gene as described in the methods, here using the sample mean and true dispersion parameter. Sample sizes of A) n = 1000 and B) n = 23 were simulated in order to understand whether the asymptotic chi square distribution was appropriate. The theoretical distributions are chi square with A) 999 degrees of freedom and B) 22 degrees of freedom. From the right hand tails we see that the observed distribution does not have values quite as extreme as those in the theoretical distribution. However, the observed distributions are very close to the theoretical distributions as demonstrated by most points lying on the identity line. We conclude that the chi-square distribution is approximately correct for the data presented herein. **Additional file****1**: **Figure S2** – Technical reproducibility and functional form of bias. Counts were scaled by total lane counts. A) Representative scatter plot of technical replicate 1 versus technical replicate 2 for one subject. Spearman correlation was 0.9941 for this pair. Axes are on the log base 2 scale. B) MVA plot for the same pair of technical replicates. The vertical axis is difference between the counts in the two replicates on the log2 scale and the horizontal axis is the average of the two counts on the log2 scale; there is one point for each gene observed in at least one replicate. The shading indicates density of points in that area with darker shading representing higher density. If two replicates yielded identical results, all points would lie on the y = 0 horizontal line (indicated on the plots for reference). A locally weighted moving average smoother is indicated to demonstrate the average bias as a function of average count. **Additional file****1**: **Figure S3** – Individual QQ plots assessing distribution of technical replicates. QQ plots for all 24 subjects for whom data was received assuming Poisson variation in pairs of technical replicates. Vertical axes indicate observed quantiles and horizontal axes indicate theoretical quantiles. Click here for file

Additional file 2 R function to plot variance as a function of the mean. Click here for file

Additional file 3 R function to create QQ plots of Pearson GOF statistics assuming the NB distribution. Click here for file
