# Supplementary material for: Technical and biological variance structure in mRNA-Seq data: life in the real world
Source: BMC Genomics. 2012 Jul 7;13:304. doi: 10.1186/1471-2164-13-304 (PMC3505161; doi:10.1186/1471-2164-13-304)

Supplementary Figures.

**Fig. Supp 1.** QQ plot of GOF statistics from simulated null (i.e., no differential expression) NB data. Data for genes were simulated with mean equal to the mean vector in the unstimulated data presented herein, dispersion parameter equal to the edgeR estimated moderated dispersion parameter values. GOF statistics were calculated for each gene as described in the methods, here using the sample mean and true dispersion parameter. Sample sizes of A) n=1000 and B) n=23 were simulated in order to understand whether the asymptotic chi square distribution was appropriate. The theoretical distributions are chi square with A) 999 degrees of freedom and B) 22 degrees of freedom. From the right hand tails we see that the observed distribution does not have values quite as extreme as those in the theoretical distribution. However, the observed distributions are very close to the theoretical distributions as demonstrated by most points lying on the identity line. We conclude that the chi-square distribution is approximately correct for the data presented herein.

A
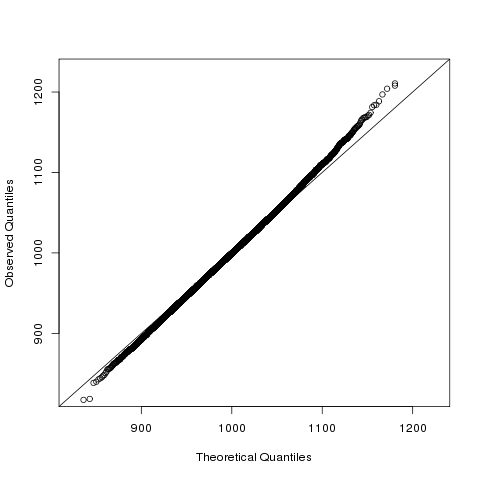
B
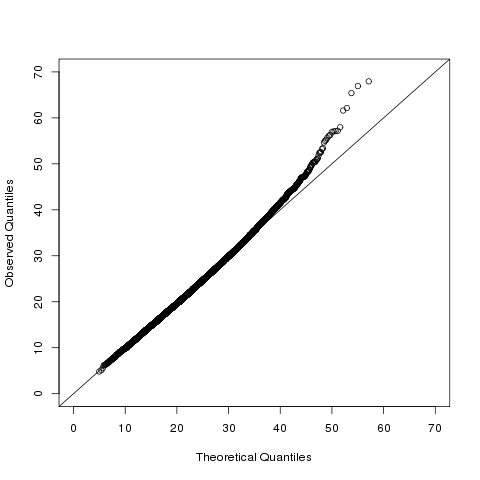


**Fig. Supp 2.** Counts were scaled by total lane counts. A) Representative scatter plot of technical replicate 1 versus technical replicate 2 for one subject. Spearman correlation was 0.9941 for this pair. Axes are on the log base 2 scale. B) MVA plot for the same pair of technical replicates. The vertical axis is difference between the counts in the two replicates on the log2 scale and the horizontal axis is the average of the two counts on the log2 scale; there is one point for each gene observed in at least one replicate. The shading indicates density of points in that area with darker shading representing higher density. If two replicates yielded identical results, all points would lie on the y=0 horizontal line (indicated on the plots for reference). A locally weighted moving average smoother is indicated to demonstrate the average bias as a function of average count.

A
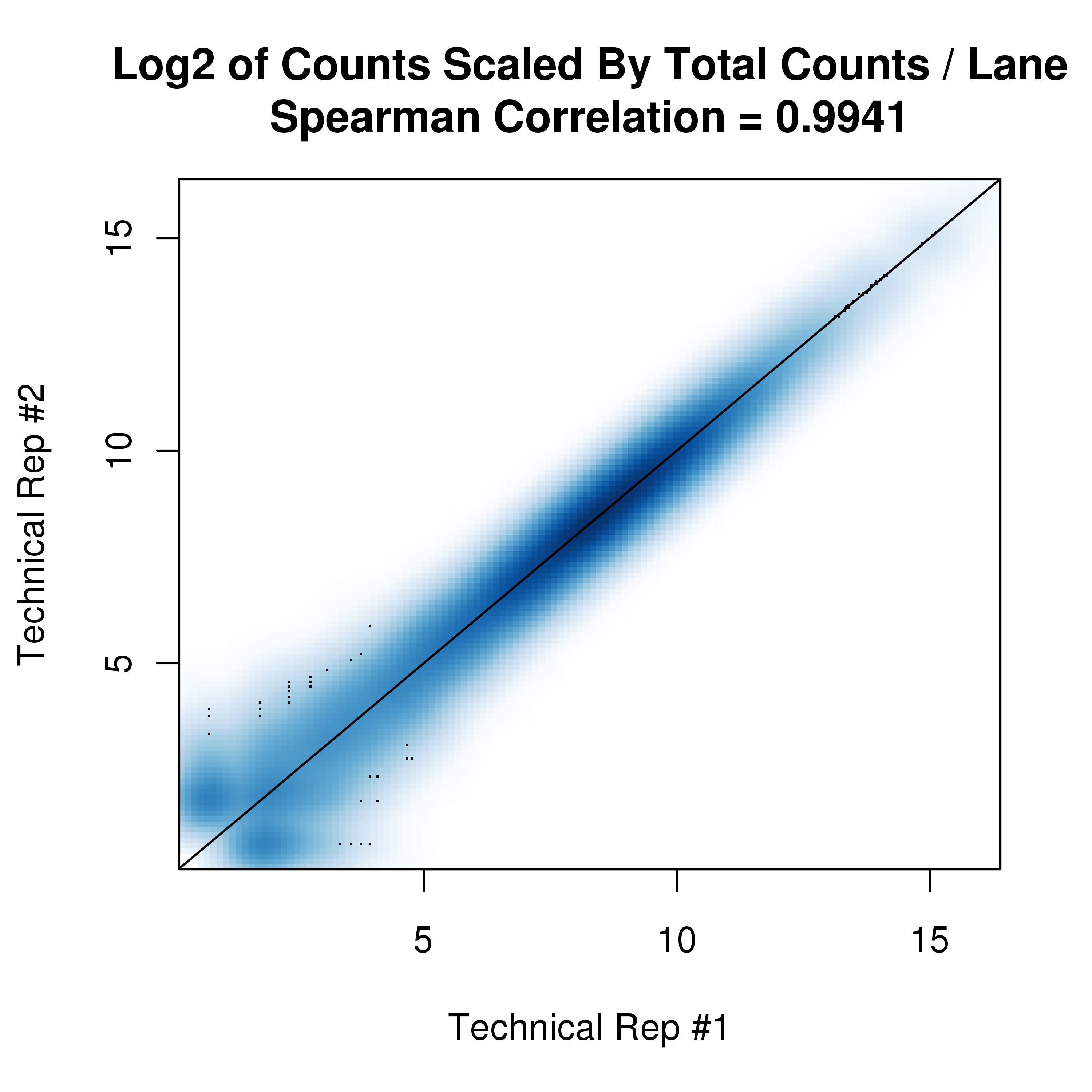
B
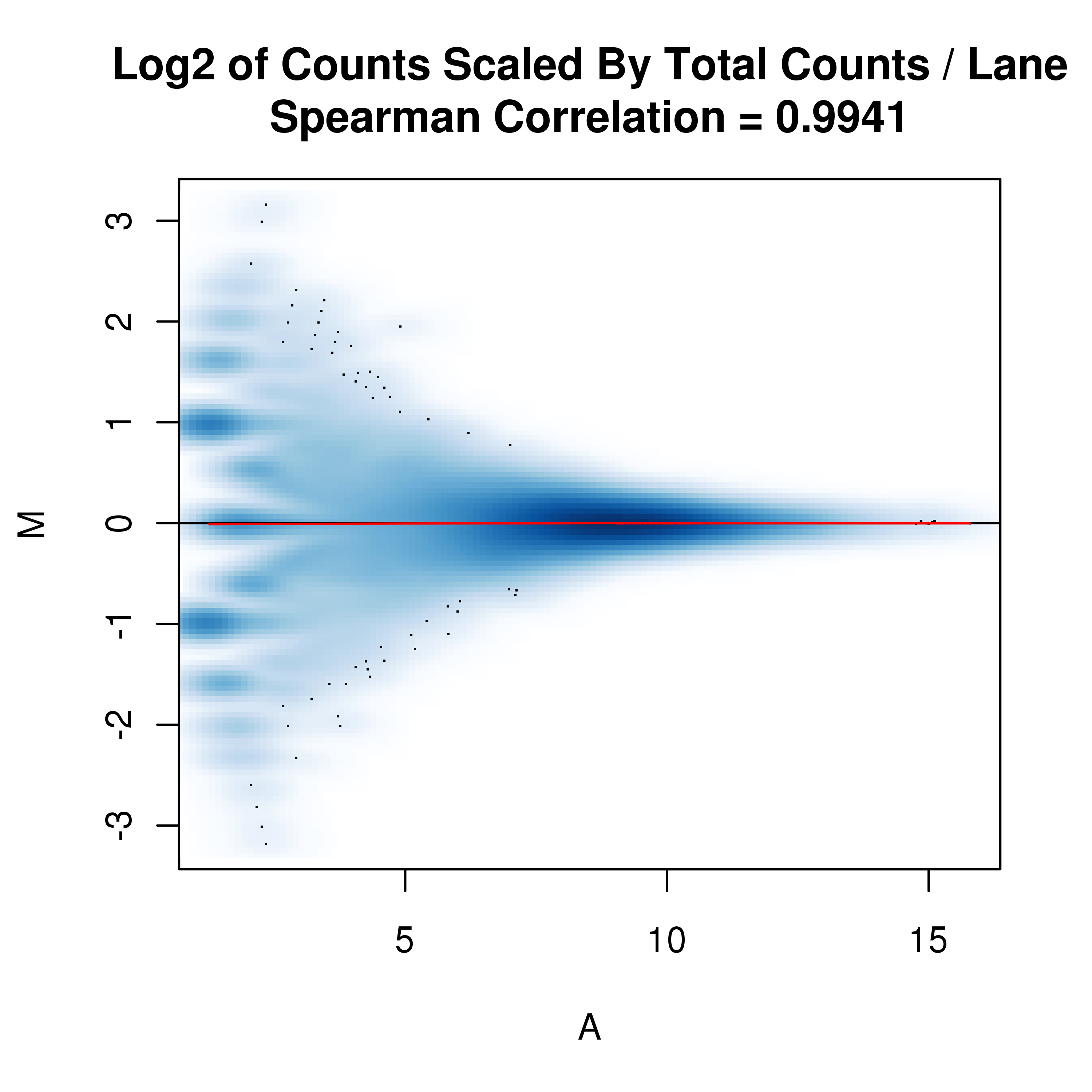


**Fig. Supp 3.** QQ plots for all 24 subjects for whom data was received assuming Poisson variation in pairs of technical replicates. Vertical axes indicate observed quantiles and horizontal axes indicate theoretical quantiles.


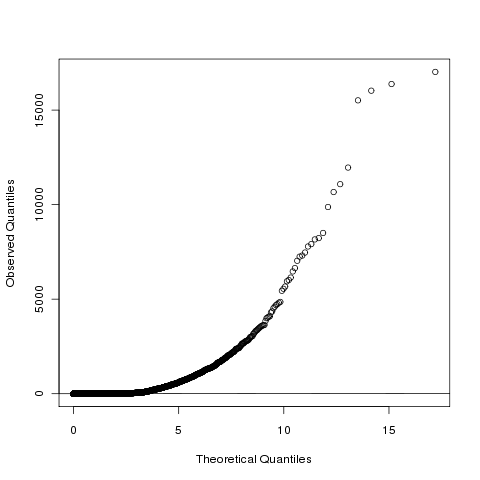


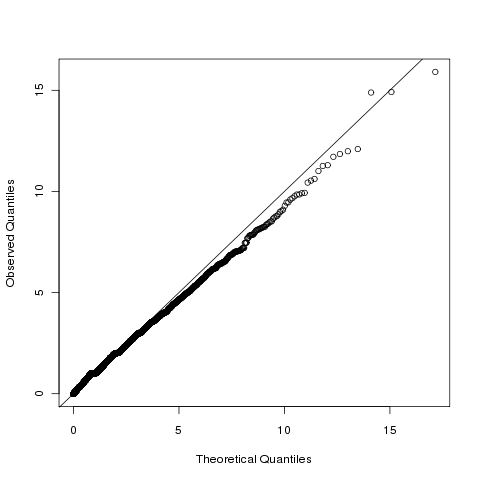

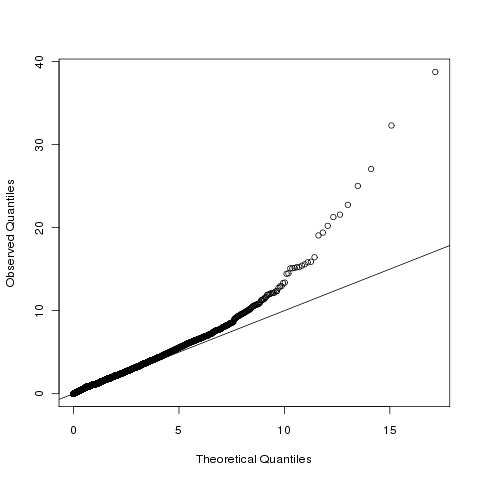

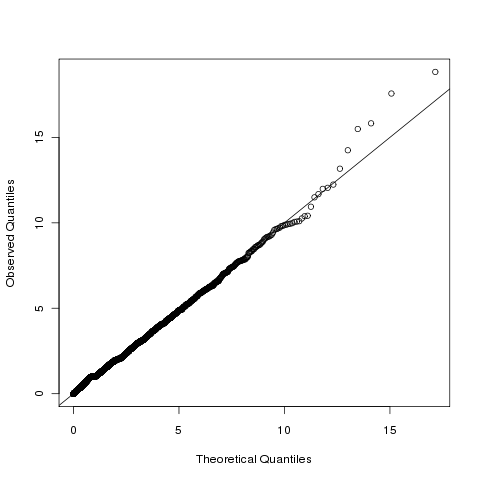

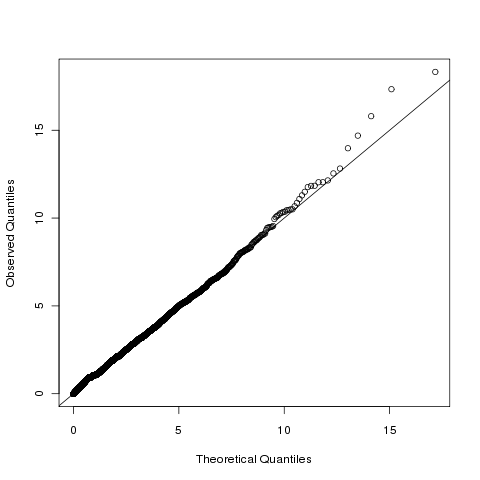

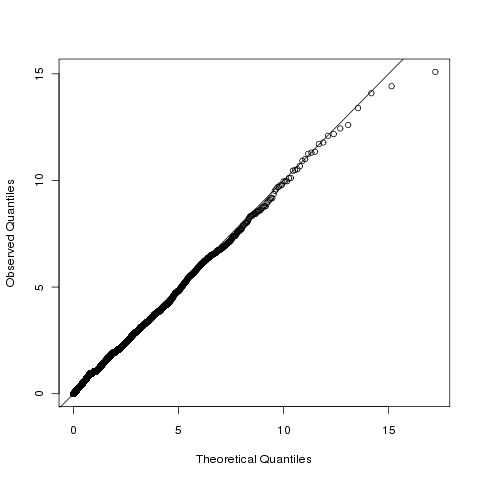

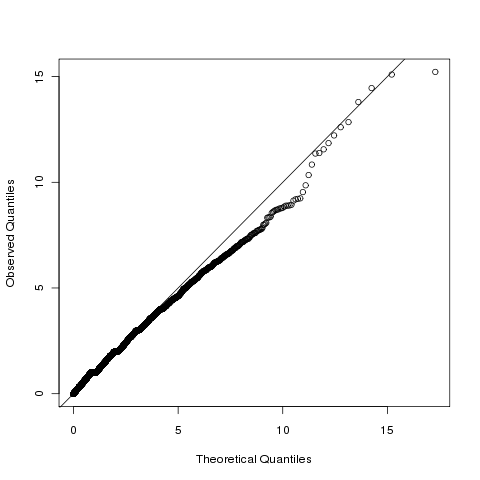

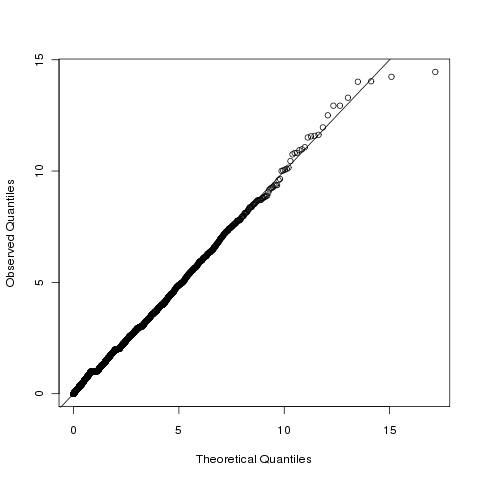

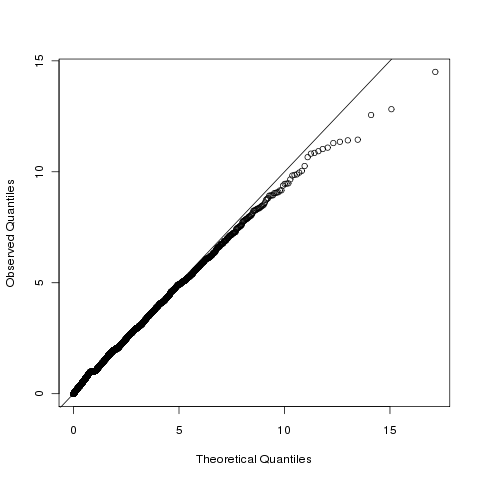

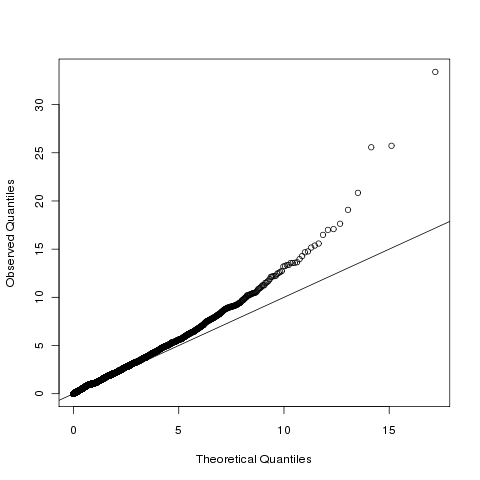

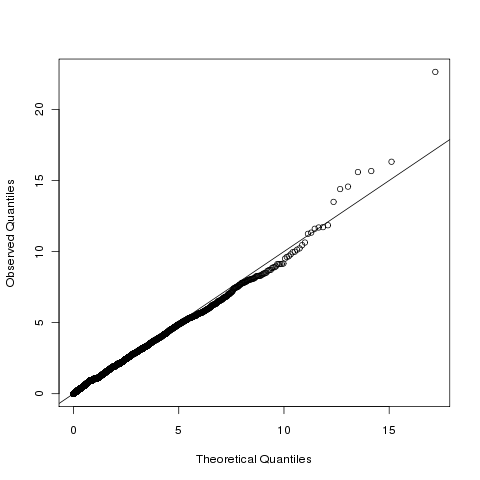

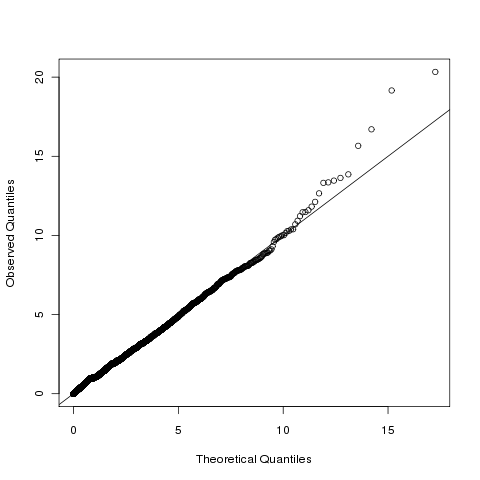

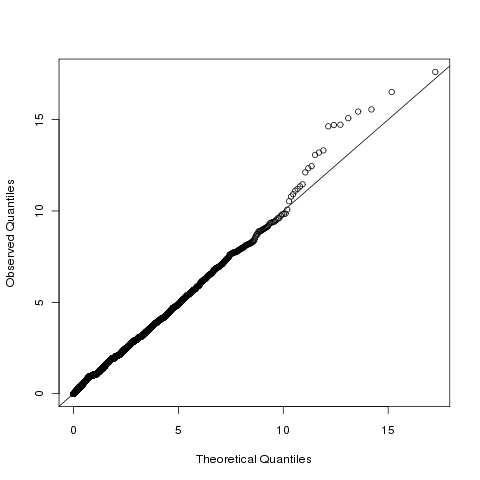

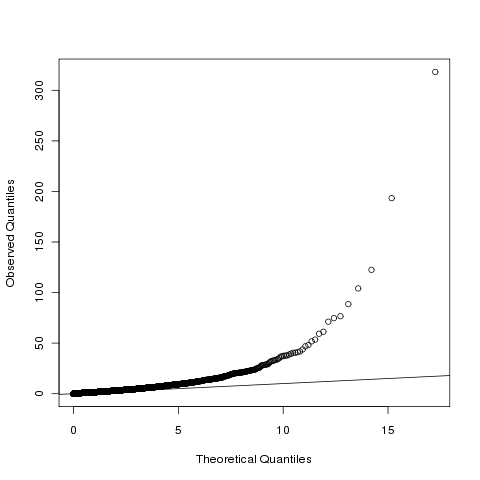

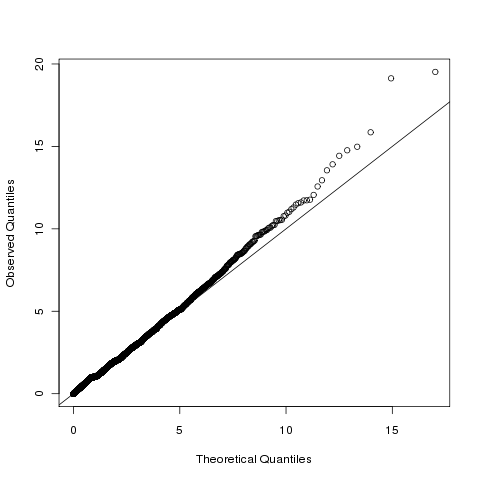

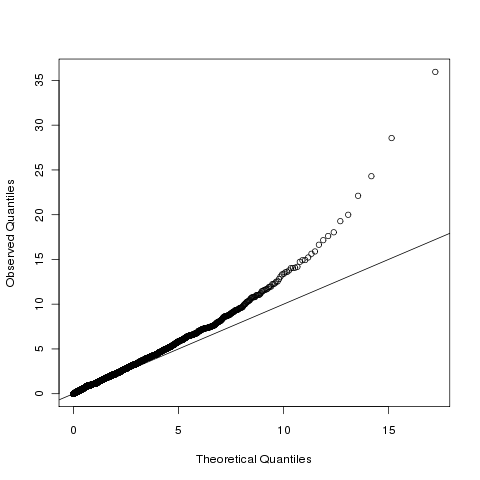

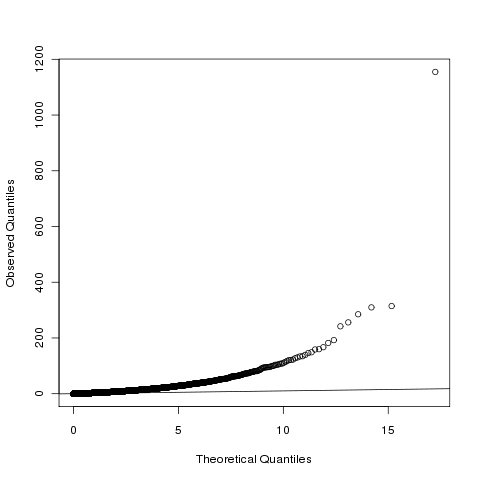

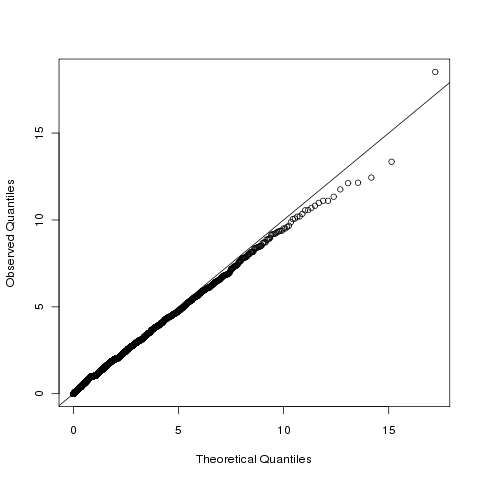

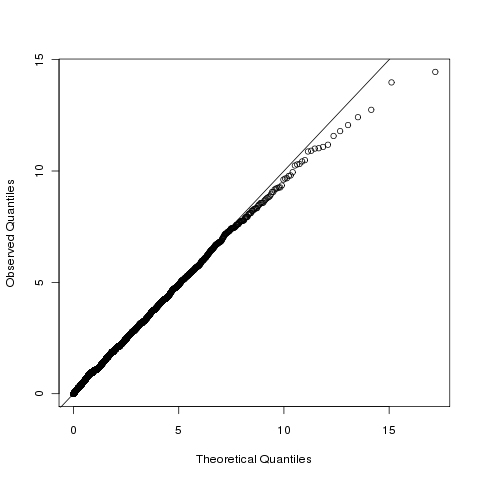

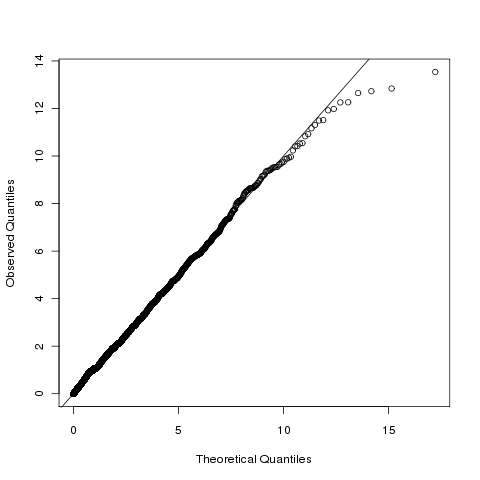

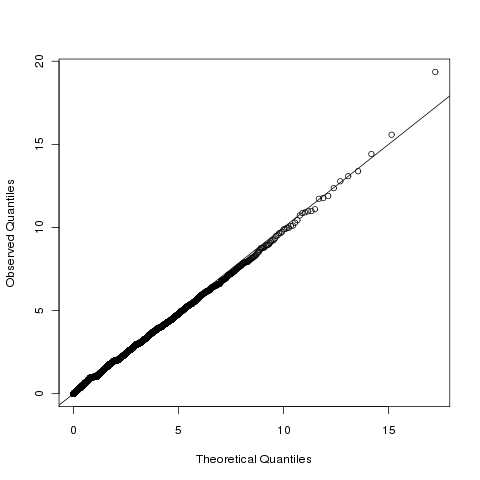

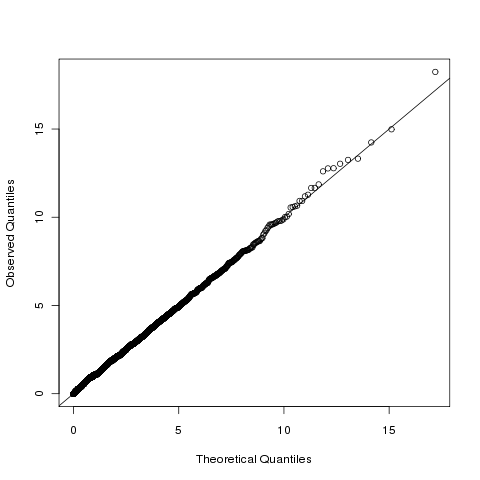

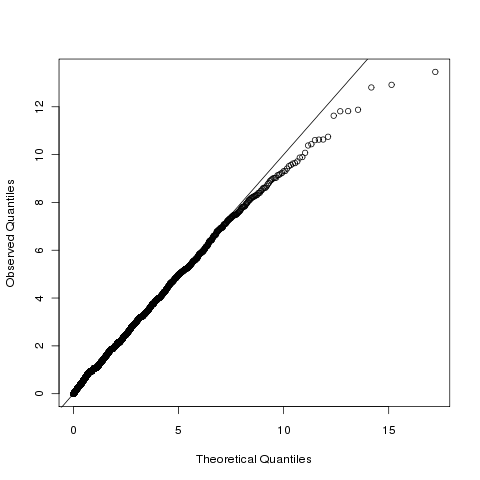

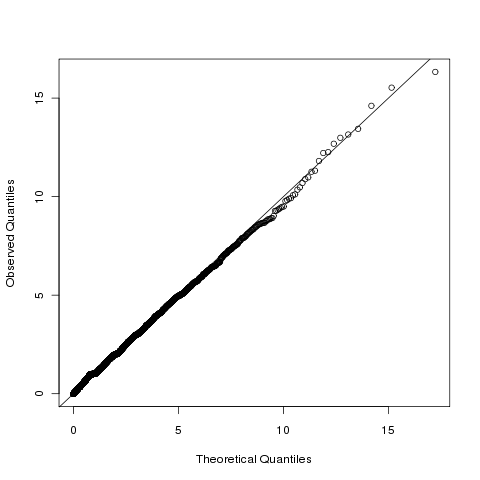

Supplement: Additional file 1 — Figure S1. Evaluation of asymptotic GOF distributional assumptions. QQ plot of GOF statistics from simulated null (i.e., no differential expression) NB data. Data for genes were simulated with mean equal to the mean vector in the unstimulated data presented herein, dispersion parameter equal to the edgeR estimated moderated dispersion parameter values. GOF statistics were calculated for each gene as described in the methods, here using the sample mean and true dispersion parameter. Sample sizes of A) n = 1000 and B) n = 23 were simulated in order to understand whether the asymptotic chi square distribution was appropriate. The theoretical distributions are chi square with A) 999 degrees of freedom and B) 22 degrees of freedom. From the right hand tails we see that the observed distribution does not have values quite as extreme as those in the theoretical distribution. However, the observed distributions are very close to the theoretical distributions as demonstrated by most points lying on the identity line. We conclude that the chi-square distribution is approximately correct for the data presented herein. Additional file1: Figure S2 – Technical reproducibility and functional form of bias. Counts were scaled by total lane counts. A) Representative scatter plot of technical replicate 1 versus technical replicate 2 for one subject. Spearman correlation was 0.9941 for this pair. Axes are on the log base 2 scale. B) MVA plot for the same pair of technical replicates. The vertical axis is difference between the counts in the two replicates on the log2 scale and the horizontal axis is the average of the two counts on the log2 scale; there is one point for each gene observed in at least one replicate. The shading indicates density of points in that area with darker shading representing higher density. If two replicates yielded identical results, all points would lie on the y = 0 horizontal line (indicated on the plots for reference). A locally weighted moving average smoo [file 1471-2164-13-304-S1.doc]
